# Les péricardites tuberculeuses au centre hospitalier universitaire de Bobo-Dioulasso, Burkina Faso

**Published:** 2012-06-01

**Authors:** Aimé Arsène Yaméogo, Carole Gilberte Kyelem, Zakari Nikiéma, Emile Birba, Téné Marceline Yaméogo, Patrice Zabsonré

**Affiliations:** 1 Institut Supérieur des Sciences de la Santé/Université Polytechnique de Bobo-Dioulasso, Burkina Faso; 2 Service de Cardiologie, Centre Hospitalier Universitaire Souro Sanou, Bobo-Dioulasso, Burkina Faso; 3 Unite de Formation et de Recherche-Science De la Santé (UFR-SDS), Université de Ouagadougou, Burkina Faso; 4 Service de cardiologie, Centre Hospitalier Universitaire Yalgado Ouédraogo, Ouagadougou, Burkina Faso

**Keywords:** Péricardite, tuberculose, insuffisance cardiaque, Cardiomégalie, Burkina-Faso

## Abstract

**Introduction:**

La tuberculose constitue toujours un problème de santé publique. Sa localisation péricardique reste fréquente. L’objectif de cette étude rétrospective descriptive était de décrire les caractéristiques cliniques et évolutives des cas de péricardites tuberculeuses dans le service de cardiologie du centre hospitalier universitaire de Bobo-Dioulasso.

**Méthodes:**

Nous avons mené une étude rétrospective descriptive des cas de péricardite tuberculeuse colligés en deux ans à partir des dossiers et registres dans le service de cardiologie du CHU de Bobo-Dioulasso de janvier 2009 à décembre 2010.

**Résultats:**

De janvier 2009 à décembre 2010, parmi 945 hospitalisations dans le service de cardiologie, une péricardite tuberculeuse a été diagnostiquée chez dix patients âgés de 18 à 82 ans. L’âge moyen était de 46,8±25 ans avec un sexe ratio de un. Soixante pour cent des patients avaient moins de 40 ans. Tous les patients avaient un niveau socio-économique bas. Une notion de contage tuberculeux a été retrouvée chez six patients. Trois patients présentaient une tuberculose pulmonaire à microscopie positive. L’insuffisance cardiaque était constante chez tous les patients avec deux cas de tamponnade à l’admission ayant nécessité une ponction péricardique d’urgence. Tous les patients avaient une sérologie VIH négative. L’échocardiographie a été importante pour le diagnostic positif et dans la prise en charge. L’évolution sous traitement antituberculeux et de l’IC a été bonne chez neuf patients à la fin de la première phase du traitement antituberculeux. Un cas de décès a cependant été enregistré chez un patient avec une HTA déjà compliquée d’accident vasculaire cérébrale ischémique.

**Conclusion:**

Les péricardiques tuberculeuses sont fréquentes au Burkina Faso. Elles touchent surtout les sujets jeunes et un intérêt particulier devrait être accordé au dépistage et au traitement précoce des cas.

## Introduction

La tuberculose constitue toujours un problème de santé publique [1]. Sa localisation pulmonaire reste de loin la plus fréquente; cependant des atteintes extra-pulmonaires notamment cardiaques sont possibles. Il s’agit le plus souvent d’une péricardite tuberculeuse pouvant être à l’origine de complications mortelles [2, 3]. Elle peut survenir à tout âge mais touche surtout le sujet jeune. L’objectif de cette étude rétrospective était de décrire les caractéristiques cliniques et évolutives des cas de péricardite tuberculeuse dans le service de cardiologie du centre hospitalier universitaire (CHU) de Bobo-Dioulasso.

## Méthodes

Nous avons mené une étude rétrospective descriptive des cas de péricardite tuberculeuse colligés en deux ans à partir des dossiers et registres dans le service de cardiologie du CHU de Bobo-Dioulasso de janvier 2009 à décembre 2010. Les paramètres étudiés ont été l’âge, le sexe les données de l’examen clinique et para clinique, notamment la radiographie pulmonaire et l’échographie cardiaque. Un examen des crachats à la recherche des bacilles acido-alcoolo résistants (BAAR) a été réalisé chez tous les patients présentant une toux chronique avec expectorations. Le diagnostic de péricardite tuberculeuse a été retenu chez tous les malades présentant des signes d’insuffisance cardiaque droite ou globale, un frottement péricardique, des signes radiologiques et écho-cardiographiques, associés à des stigmates biologiques indirects de tuberculose (liquide séro-fibrineux, hyper-lymphocytose) dans les liquides de ponction péricardique et d’ascite.

## Résultats

Durant les 24 mois d’étude, 945 patients ont été hospitalisés dans le service de cardiologie. Un diagnostic de péricardite tuberculeuse a été retenu chez dix patients âgés de 18 à 82 ans. L’âge moyen était de 46,8±25 ans avec un sex ratio de un. Parmi ces cas, 60% des patients avaient moins de 40 ans. Tous les patients avaient un niveau socio-économique bas. Une notion de contage tuberculeux a été retrouvée chez six patients. Un patient était porteur d’une HTA déjà compliquée d’un accident vasculaire cérébral (AVC) ischémique. Les patients présentaient plusieurs signes associés résumés sur le Tableau 1.

**Tableau 1:** Répartition de dix cas de péricardite tuberculeuse selon les signes cliniques radiographiques et écho-cardiographiques

Signes cliniques	Effectifs	%
Toux	9	90
Fièvre vespérale	6	60
Amaigrissement	9	90
Sueurs nocturnes	2	20
Hémoptysie	2	20
Douleurs thoracique	8	80
Dyspnée	10	100
Frottement péricardique	5	50
Tachycardie	6	60
Tamponnade	2	20
Ascite	4	40
Insuffisance cardiaque globale	6	60
Insuffisance cardiaque droite	4	40
**Echocardiographie**		
Epanchement péricardique	6	60
**Radiographie pulmonaire**		
Cardiomégalie	7*	70
Miliaires	2	20
Cavernes tuberculeuse	1	10

* (Index Cardio-Thoracique moyen (ICT) à 0,75 extrêmes 0,60 – 0,90)

**Figure 1:**
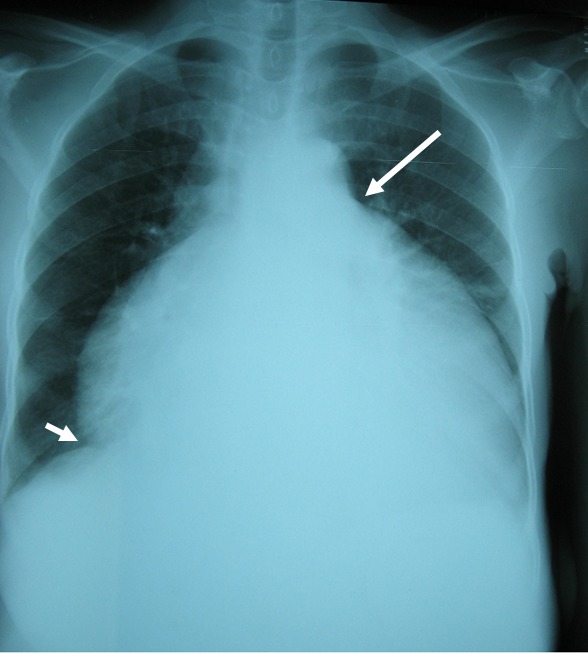
Cardiomégalie globale avec disparition des arcs, un pédicule court et angle cardio phrénique droit ouvert chez une patiente de 70 ans (ICT = 0,88)

**Figure 2:**
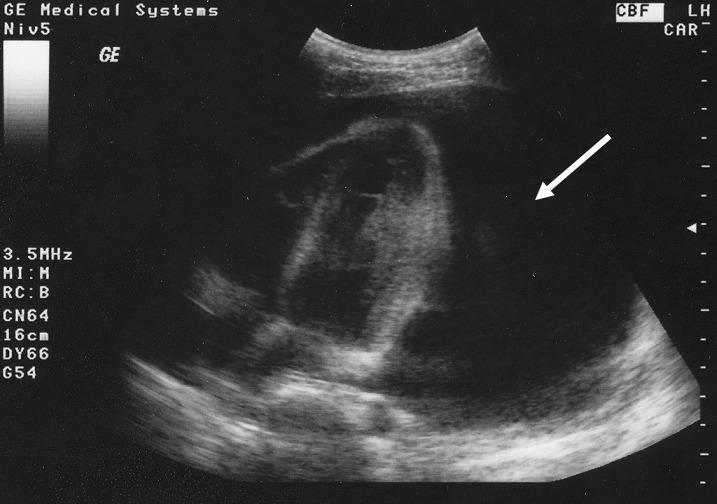
Important épanchement péricardique (flèche) à liquide citrin chez une patiente de 18 ans

**Figure 3:**
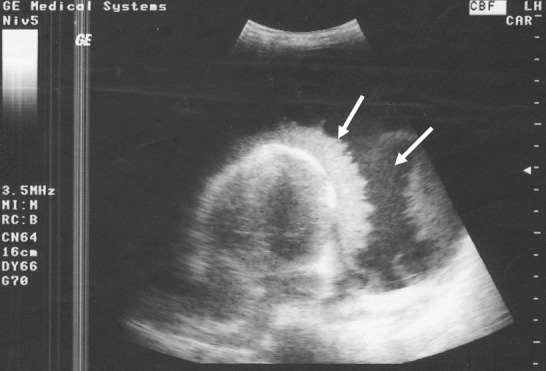
Epanchement péricardique purulent avec amas de fibrine (flèches) chez une patiente de 17 ans

Trois patients présentaient une tuberculose pulmonaire à microscopie positive (TPM + ), trois autres une tuberculose pulmonaire à microscopie négative (TPM-). Dans 70% des cas il s’agissait d’une atteinte de plusieurs séreuses avec des adénopathies médiastinales et abdominales objectivées à la radiographie pulmonaire et à l’échographie abdominale. Deux patients présentaient une tamponnade à leurs admissions ayant nécessité une ponction péricardique en urgence. Elle a ramené respectivement 2160 et 1400 cc de liquide citrin. Les cas de tamponnade et les épanchements péricardiques de plus de 3 mm à l’échographie, ont fait l’objet d’une ponction péricardique ramenant parfois du liquide purulent après deux ou trois ponctions, compte tenu de la rapidité de reconstitution de l’épanchement et de l’absence de drain péricardique (Figure 1, Figure 2 et Figure 3). Aucun patient de notre série ne présentait une sérologie HIV positive.

Tous les patients ont bénéficié du traitement spécifique de la tuberculose associé à un traitement classique de l’insuffisance cardiaque quand cela était nécessaire. L’évolution clinique en hospitalisation a été bonne chez neuf patients avec une régression totale des signes cliniques à la fin de la première phase du traitement anti tuberculeux. Un cas de décès a été enregistré dans notre série. Il s’agissait d’un cas avec une hypertension déjà compliquée d’AVC ischémique.

## Discussion

La tuberculose reste toujours une pathologie fréquente et grave. Sa localisation extra-pulmonaire dans la population générale serait de l’ordre de 15% [4]. Des complications cardiaques sont possibles mais l’atteinte péricardique est de loin la plus fréquente [5]. D’environ 1 à 8% de prévalence dans la littérature générale selon Sida-Diaz [1]; dans les pays en voie de développement comme le nôtre, la tuberculose constitue la première cause de péricardite [6]. Cette prévalence pourrait atteindre 65% sur terrain VIH [1, 5]. Une étude faite par Lepori [7] a d’ailleurs montré une augmentation des cas de péricardite en Afrique, en relation avec celle des cas de Sida [7]. La péricardite tuberculeuse touche surtout les sujets jeunes de moins des 40 ans (60% dans notre série); ce qui est conforme aux données de la littérature [5, 6].

La suspicion diagnostic de péricardite tuberculeuse devrait être clinique devant des signes classiques dits d’imprégnation tuberculeuse associés à une toux chronique très souvent sèche, des douleurs thoraciques précordiales et un syndrome d’épanchement péricardique. En effet, elle se caractérise par l’abondance de l’épanchement péricardique souvent circonférentiel qui peut rapidement mettre en jeu le pronostic vital par une tamponnade [8, 9]. Une étude Sud-Africaine sur l’épidémiologie des épanchements péricardiques avait montré que dans 69,5% des cas, les épanchements de grande abondance étaient d’origine tuberculeuse [5]. L’échocardiographie bidimensionnelle reste essentielle pour le diagnostic positif car le tableau clinique peut être insidieux avec une tamponnade inaugurale [1, 3, 10].

Cependant, le diagnostic étiologique pose souvent problème [8]. Seuls trois patients dans notre série se sont révélés être des TPM+. Le diagnostic de tuberculose chez les autres patients qui présentaient une atteinte des séreuses l’a été sur la base des arguments épidémiologiques, cliniques, échographiques et de signes indirects de tuberculose dans les liquides de ponction péricardique.

L’évolution sous traitement est un argument de valeur dans la prise en charge de la tuberculose dans notre contexte de travail comme dans les séries africaines [6]. Ceci relance la question de la prévention de tuberculose et surtout des primo infections tuberculeuses. L’intradermo-réaction à la tuberculine peu pratiquée de nos jours au Burkina Faso devrait reprendre sa place dans le diagnostic de la tuberculose. Les récentes recommandations de la Mayo Clinic aux USA [11] révèlent d’ailleurs que les primo infections tuberculeuses devraient faire l’objet d’un traitement à l’Isoniazide pendant neuf mois et les formes actives du traitement habituel afin d’éviter l’émergence des résistances. Par ailleurs, il semble que les épanchements des péricardites tuberculeuses devraient être drainés du fait de leur abondance et des reconstitutions rapides [8], les ponctions itératives étant une source de surinfections.

Le pronostic dépend de la rapidité du diagnostic. En effet il faut toujours redouter la péricardite chronique constrictive. Imazio [12] rapportait dans une étude d’une cohorte de 500 cas de péricardite un taux de constriction de 31,65 p. 1000 pour les péricardites tuberculeuses. Ce taux pouvant atteindre 52,74 p.1000 lorsqu’elles devenaient purulentes.

## Conclusion

Les complications péricardiques de la tuberculose sont fréquentes au Burkina Faso. Si l’échocardiographie est incontournable pour le diagnostic positif, le diagnostic étiologique lui pose toujours problème dans notre contexte de travail. Le traitement devrait être entrepris sur la base des données cliniques, biologiques et échographiques afin d’éviter les complications aigues souvent mortelles. Le drainage péricardique devrait être plus souvent effectué du fait de la reconstitution rapide de l’épanchement. Un intérêt particulier devrait être accordé au dépistage en zone urbaine mais surtout rurale pour une prise en charge précoce et adéquate chez les sujets jeunes.
